# Association of IMP3 and CD10 Expression with Clinicopathological Features and Outcomes in Phyllodes Tumors: A Retrospective Single-Center Study

**DOI:** 10.3390/jcm15041614

**Published:** 2026-02-19

**Authors:** Hülya Odabaşı Bükün, Erdem Çubukçu, Adem Deligönül, Ahmet Bilgehan Şahin, Alper Coşkun, Gül Akın, Çağla Karaoğlu, Ali Aktaş, İlkay Gönül, Mine Özşen, Seyit Ali Volkan Polatkan, Türkkan Evrensel

**Affiliations:** 1 Department of Medical Oncology, Uludag University, 16100 Bursa, Türkiye; erdemcubukcu@uludag.edu.tr (E.Ç.); ademd@uludag.edu.tr (A.D.); absahin@uludag.edu.tr (A.B.Ş.); alpercoskun@uludag.edu.tr (A.C.); gulakin@uludag.edu.tr (G.A.); caglakaraoglu@uludag.edu.tr (Ç.K.); aliaktas@uludag.edu.tr (A.A.); evrensel@uludag.edu.tr (T.E.); 2 Department of Internal Medicine, Uludag University, 16100 Bursa, Türkiye; ilkaygonul@uludag.edu.tr; 3 Department of Pathology, Uludag University, 16100 Bursa, Türkiye; mineozsen@uludag.edu.tr; 4 Department of General Surgery, Uludag University, 16100 Bursa, Türkiye; volkanpolatkan@uludag.edu.tr

**Keywords:** phyllodes tumor, IMP3, CD10, prognosis, immunohistochemistry, survival, breast neoplasm, risk stratification, stromal biomarkers

## Abstract

**Background/Objectives:** Phyllodes tumors (PTs) are rare fibroepithelial breast neoplasms with highly variable clinical behavior. Identifying predictive immunohistochemical markers is crucial for early detection of lesions with malignant potential and appropriate treatment selection. This study aimed to evaluate the association of insulin-like growth factor II mRNA-binding protein 3 (IMP3) and cluster of differentiation 10 (CD10) expression with histopathological grade and survival outcomes in PTs. **Methods:** We retrospectively analyzed sixty-eight female patients with PTs at Uludag University Faculty of Medicine Hospital, between 2000 and 2024. Histopathological features, IMP3 and CD10 expression, and clinical outcomes were evaluated. Disease-free survival (DFS) and overall survival (OS) were estimated using the Kaplan–Meier method and compared using the log-rank test. **Results:** Among the 68 patients (median age: 39.0 years), 60.3% had benign PTs, 14.7% had borderline PTs, and 25.0% had malignant PTs. Histopathological parameters differed significantly across grades (all *p* < 0.01). IMP3 expression was strongly associated with higher histological grade, larger tumor size, and stromal atypia (*p* < 0.05). OS differed significantly by histological grade (*p* = 0.009) and IMP3 status (*p* = 0.013), whereas DFS and CD10 expression showed no significant associations (*p* > 0.05). **Conclusions:** IMP3 expression is strongly associated with malignant histology and poorer overall survival in PTs; however, its independent prognostic value could not be conclusively established due to the limited number of outcome events. While IMP3 may serve as a promising marker in routine pathological assessment, validation in larger, prospective cohorts with sufficient event numbers is warranted.

## 1. Introduction

Phyllodes tumors (PTs) are rare fibroepithelial breast neoplasms representing less than 1% of all breast tumors [1,2]. They arise from the stromal component of the breast and are classified as benign, borderline, or malignant based on histological criteria [3,4,5]. Their clinical behavior is highly variable, ranging from indolent lesions to aggressive tumors capable of local recurrence or distant metastasis. Accurate grading is therefore essential for determining surgical management and follow-up strategies [6,7,8]. However, histological grade alone does not fully capture the biological heterogeneity of PTs, particularly within borderline and malignant categories, where tumors with similar histological features may demonstrate markedly different clinical outcomes.

Malignant PTs typically exhibit marked stromal atypia, stromal overgrowth, high mitotic activity (≥10 mitoses per 10 high-power fields [HPF]), infiltrative borders, and increased cellularity [9]. Although wide local excision remains the primary treatment, the optimal margin width and the role of adjuvant therapies remain controversial due to limited high-level evidence [6]. This uncertainty underscores an unresolved prognostic gap in contemporary clinical practice, highlighting the need for additional biomarkers that may improve risk stratification beyond conventional histopathological assessment.

Previous studies have demonstrated that several key proteins—including S100A8, Ki-67, polycomb repressive complex 2 (PRC2) and its catalytic subunit enhancer of zeste homolog 2 (EZH2), trimethylation of lysine 27 at histone H3 (H3K27me3), and programmed death-ligand 1 (PD-L1)—play important roles in the development and progression of breast fibroepithelial lesions. Accordingly, identification of these molecules may be useful in differentiating PT histologies [10,11,12,13]. Nevertheless, many of these markers have shown variable reproducibility across studies, and their prognostic utility has often been inferred from associations with histological grade rather than validated survival endpoints. Given the well-recognized diagnostic and prognostic challenges associated with PTs, there is growing interest in the application of immunohistochemical markers targeting these pathways.

In addition, several immunohistochemical markers related to the tumor microenvironment—particularly tumor-associated macrophages (TAMs)—including CD34, CD68, CD80, CD86, CD163, CD204, and CD206, as well as markers such as β-catenin, GATA3, TRPS1, cytokeratins, Ki-67, p63, amine oxidase-related proteins, epidermal growth factor receptor (EGFR), and somatostatin receptors, have been implicated in either tumor-suppressive or tumor-promoting processes [11,13,14,15,16,17]. These molecules reflect diverse biological mechanisms such as stromal–epithelial interaction, immune modulation, proliferation, and differentiation, all of which may contribute to PT behavior. Despite this expanding body of literature, the clinical applicability of many proposed biomarkers remains limited by small cohort sizes, heterogeneous study designs, and inconsistent associations with recurrence or survival outcomes.

Cluster of differentiation 10 (CD10), a stromal zinc-dependent metalloendopeptidase, has been shown to aid in distinguishing PTs from fibroadenomas and has also been associated with more aggressive clinical behavior [16,18,19]. Insulin-like growth factor II mRNA-binding protein 3 (IMP3), an oncofetal RNA-binding protein, exhibits strong stromal expression particularly in malignant and recurrent PTs and is thought to reflect increased biological aggressiveness [20,21]. However, existing studies evaluating CD10 and IMP3 in PTs are largely retrospective, often include limited patient numbers, and frequently lack long-term survival analyses, resulting in uncertainty regarding their true prognostic relevance. Moreover, data directly linking these markers to disease-free survival (DFS) and overall survival (OS) remain sparse and inconsistent.

Based on their diagnostic and prognostic relevance in mesenchymal tumors, we hypothesized that higher expression levels of CD10 and IMP3 correlate with adverse outcomes in patients with PTs. However, evidence regarding their prognostic significance—especially in relation to long-term survival—remains limited, as most available studies are constrained by small sample sizes and lack comprehensive survival analyses. Therefore, the aim of this study was to evaluate the association of IMP3 and CD10 expression with histological grade, clinicopathological features, DFS, and OS in a cohort of 68 female patients with PTs.

## 2. Materials and Methods

### 2.1. Study Design and Patient Selection

This study was conducted as a retrospective cohort analysis. All tissue specimens consisted of formalin-fixed, paraffin-embedded (FFPE) samples originally obtained for routine diagnostic purposes, and no additional tissue sampling or patient intervention was performed for research purposes. Patients diagnosed with PTs at the Department of Medical Oncology, Uludag University Faculty of Medicine Hospital, between January 2000 and December 2024 were included. Demographic information, clinical characteristics, and treatment details were obtained retrospectively from electronic medical records and archival files.

Inclusion criteria were as follows: (1) histopathologically confirmed primary breast PT using tissue blocks in the pathology archive; (2) adequate tumor sampling defined as the procurement of at least one tissue block for each 1-cm increment of the maximum tumor diameter, in accordance with the pathology team’s standardized sampling protocol; and (3) minimum follow-up duration of 6 months.

**Figure 1:**
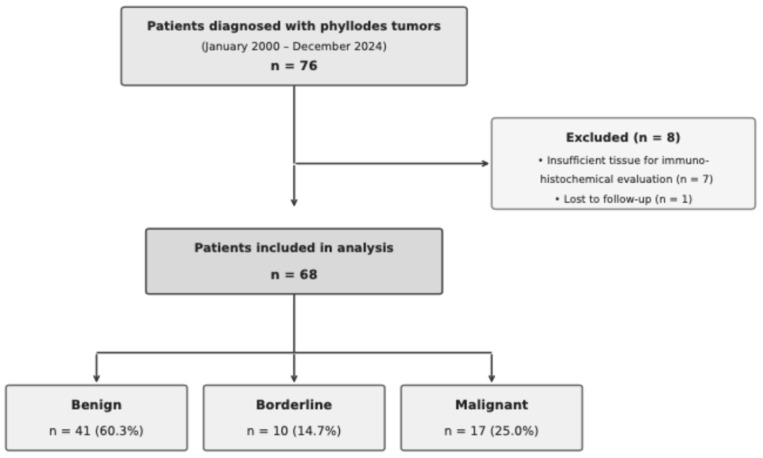
Patient selection flowchart. Flow diagram illustrating the selection process of patients with phyllodes tumors (PTs) diagnosed at Uludag University Faculty of Medicine Hospital between January 2000 and December 2024. The final cohort included 68 female patients meeting all inclusion criteria.

Exclusion criteria were as follows: (1) male sex; (2) secondary or recurrent breast sarcoma; (3) insufficient tissue quantity or quality for immunohistochemical evaluation; (4) prior history of another primary breast malignancy or other types of malignancy; and (5) incomplete clinicopathological data (Figure 1).

### 2.2. Histopathological Examination

Hematoxylin and eosin-stained slides from all cases were independently re-evaluated by an experienced pathologist with at least 10 years of experience in breast pathology and classified as benign, borderline, or malignant PTs according to the 2019 World Health Organization (WHO) classification of breast tumors. Classification included stromal cellularity (mild, moderate, or marked), stromal atypia (mild, moderate, or severe), mitotic activity (<5, 5–9, or ≥10 per 10 HPF), stromal overgrowth (present or absent), and tumor borders (circumscribed versus infiltrative). Benign tumors exhibited mild stromal cellularity, absent or mild atypia, fewer than 5 mitoses per 10 HPF, no stromal overgrowth, and circumscribed borders [22]. In addition, the Ki-67 proliferation index, expressed as the percentage of positively stained stromal nuclei, was assessed by immunohistochemical staining as a supportive proliferative marker [11,23].

### 2.3. Immunohistochemical Evaluation

Immunohistochemical staining was performed on 4-μm sections obtained from archival paraffin blocks. Antigen retrieval was performed using CC1 Solution (pH 9) by incubating the sections at 97 °C for 60 min. Subsequently, endogenous peroxidase activity was blocked using the UV Inhibitor included in the secondary detection kit. Primary antibodies against IMP3 (clone EP286, catalog no. 433R-14, Cell Marque, Rocklin, CA, USA) and CD10 (clone SP67, catalog no. R0585578560001 Roche, Tuscon, AZ, USA) were applied, with 2 h (1:25 dilution) and 1 h incubation, respectively. In addition, estrogen receptor (ER, clone SP1; catalog no. N19436; Roche Diagnostics), progesterone receptor (PR, clone 1E2; catalog no. N13902; Roche Diagnostics), and Ki-67 (clone 30-9; catalog no. N10219; Roche Diagnostics) antibodies were used. For secondary detection, the ROCHE UltraView Universal DAB Detection Kit was used following the standard UltraView IHC protocol. All procedures were performed on the ROCHE Ventana BenchMark Ultra IHC Stainer (Roche/Ventana, Tucson, AZ, USA).

**Figure 2:**
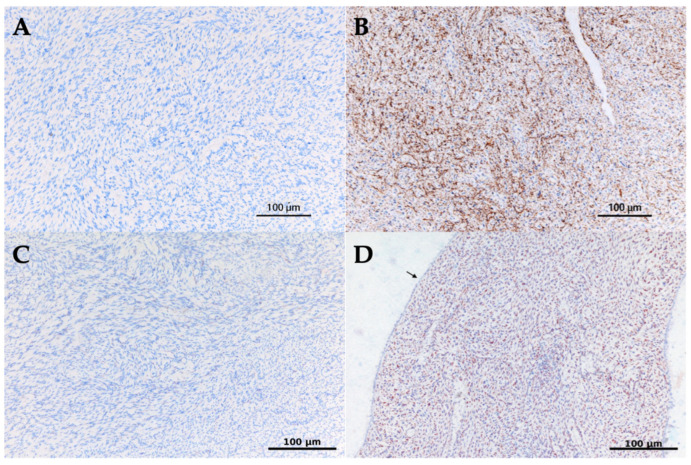
Representative immunohistochemical staining patterns for IMP3 and CD10 in PTs. (**A**) Borderline phyllodes tumor: No CD10 immunoreactivity is observed in the stromal component. (**B**) Malignant phyllodes tumor: Diffuse CD10 immunoreactivity is observed in the stromal component. (**C**) Borderline phyllodes tumor: No IMP3 immunoreactivity is detected in the stromal component. (**D**) Malignant phyllodes tumor: Stromal cells show positive IMP3 immunoreactivity, whereas the epithelial component is negative (epithelial component indicated by arrow). All images: original magnification ×200; scale bars = 100 μm. Abbreviations: IMP3, insulin-like growth factor II mRNA-binding protein 3; CD10, cluster of differentiation 10.

IMP3 staining was evaluated as cytoplasmic positivity in the stromal component, and staining in more than 10% of stromal cells was considered positive [21]. CD10 staining exhibited membranous and/or cytoplasmic patterns in stromal cells, and staining in more than 20% of cells was classified as positive [4]. ER and PR were evaluated as nuclear positivity in the epithelial component, and 1% or greater nuclear staining was considered positive. All immunohistochemical evaluations were performed by pathologists blinded to clinical outcomes and independent of the study hypothesis (Figure 2).

### 2.4. Endpoints

The primary endpoints were DFS and OS. DFS was defined as the time from surgery to documented local or distant recurrence, or death from any cause, whichever occurred first. OS was defined as the time from the date of diagnosis to death from any cause. Patients without events at last follow-up were censored at the date of last contact.

### 2.5. Follow-Up Procedures

Patients were followed according to a standardized institutional follow-up protocol routinely applied in clinical practice, which remained consistent throughout the study period. Follow-up data—including clinical examinations, laboratory tests, and imaging studies (chest computed tomography [CT] and breast magnetic resonance imaging [MRI])—were retrieved retrospectively from medical records. According to this protocol, patients underwent evaluation every 3 months during the first 2 years, every 6 months between years 3 and 5, and annually thereafter. No additional prospective follow-up visits or investigations were performed specifically for the purposes of this study.

A positive surgical margin was defined as the presence of neoplastic tissue in direct continuity with the inked resection margin on histopathological examination. Surgical margins were considered negative when no tumor cells were identified at the inked surface [24].

Recurrence was defined as the detection of tumor reappearance after initial surgical treatment and was classified as local or distant. Local recurrence was identified by radiological evidence of tumor at or adjacent to the primary surgical site, with or without histopathological confirmation when clinically indicated. Distant recurrence was defined as radiological and/or histopathological evidence of tumor involvement at sites anatomically distinct from the primary tumor location [25].

Clinical and pathological data were retrieved retrospectively from institutional medical records and pathology archives and were fully anonymized prior to analysis.

### 2.6. Sample Size

A post hoc power analysis was performed to evaluate the statistical adequacy of the sample size for the association between IMP3 expression and histological grade. The observed effect size was found to be large (Cramér’s V = 0.648), yielding an achieved power of 99.9% with a sample of 68 patients. Power calculations were based on observed effect sizes using Cohen’s w statistic and chi-square distribution with α = 0.05. No post hoc analysis was performed for the survival outcomes.

### 2.7. Statistical Analysis

Statistical analyses were performed using Jamovi (Version 2.6.44; The Jamovi Project, 2023) and JASP (Version 0.19.3; Jeffreys’ Amazing Statistics Program, 2024). Survival analyses and Cox regression models were conducted using R software (version 4.5.2; R Foundation for Statistical Computing, Vienna, Austria). A two-sided *p* value of 0.05 or less was considered statistically significant.

The distribution of continuous variables was assessed using the Shapiro–Wilk test and inspection of histograms. Variables not following a normal distribution were summarized as median [minimum–maximum], whereas normally distributed variables were presented as mean ± standard deviation (SD).

Comparisons of demographic and clinicopathological characteristics across histological grade groups (benign, borderline, and malignant) were performed using the Kruskal–Wallis H test for continuous variables and Pearson’s chi-square test or Fisher–Freeman–Halton exact test for categorical variables, depending on expected cell counts. When omnibus tests indicated statistically significant differences, pairwise comparisons were conducted using Bonferroni-corrected Mann–Whitney U tests for continuous variables and Bonferroni-adjusted z-tests for proportions in categorical variables. In post hoc analyses, statistically distinct subgroups within each variable category were indicated with superscript letters (a, b, c).

DFS and OS were estimated using the Kaplan–Meier method. DFS and OS curves were compared according to pathological diagnosis (benign, borderline, and malignant) and CD10/IMP3 expression status using the log-rank test. Survival curves and event counts were calculated using the survival package in R (functions Surv, survfit, and survdiff), and graphical representations were generated using the survminer package.

A *p* value less than 0.05 was considered statistically significant, whereas a *p* value between 0.05 and 0.10 was interpreted as borderline (marginal) significance in the context of a small sample size and low event rate.

### 2.8. Ethical Approval

This study was conducted in accordance with the Declaration of Helsinki and relevant national regulations governing retrospective research in Türkiye. The secondary use of archived FFPE tissue samples, originally obtained for routine diagnostic purposes, is permitted under Turkish regulations provided that no additional patient intervention is performed and that patient data are anonymized (Regulation on Clinical Trials of Medicinal Products and Biological Products, Official Gazette No. 28617, 2013).

The study was approved by the Health Research Ethics Committee of Uludag University Faculty of Medicine (Decision No: 2025/538-6/2; Date: 3 March 2025). Owing to the retrospective design and the use of archived diagnostic material, the requirement for informed consent was waived.

## 3. Results

A total of 68 female patients with median age of 39.0 years [range: 13.9–74.0] were included in the study. Of these, 60.3% (*n* = 41) had benign, 14.7% (*n* = 10) had borderline, and 25.0% (*n* = 17) had malignant histological subtypes. The median follow-up time was 83.2 months (range: 3.2–166.9 months). Although patients with malignant tumors were older (median age: 46.3 years), this difference did not reach statistical significance (*p* = 0.054). The median tumor size was 5.0 cm (range: 1.0–14.0 cm). Although larger tumors were prominent in the malignant group, this difference was not statistically significant compared to other groups. No significant differences were observed among groups regarding surgical approach or positive surgical margins (*p* > 0.05). Analysis of histopathological parameters demonstrated significant differences in mitotic activity distribution among groups (*p* = 0.005). High mitotic activity was observed in 80.0% of malignant tumors but was absent in borderline tumors. Stromal atypia was present in 82.4% of malignant tumors compared to approximately 22.0% in benign and borderline groups (*p* < 0.001). Median Ki-67 proliferation index values were 25.0% in malignant, 15.0% in borderline, and 10.0% in benign tumors (*p* < 0.001) (Table 1).

Evaluation of immunohistochemical markers revealed a strong association between IMP3 positivity and histological grade (*p* < 0.001). IMP3 positivity was observed in 76.5% of malignant tumors, 30.0% of borderline tumors, and 7.3% of benign tumors, with all three groups significantly different from each other. The difference in CD10 expression among groups did not reach statistical significance (*p* = 0.065). No significant differences were observed regarding hormone receptor positivity (*p* > 0.05) (Table 1).

Regarding treatment modalities, adjuvant chemotherapy was more frequent in the malignant group. Postoperative radiotherapy was administered to 58.8% of patients with malignant tumors compared with approximately 20% in benign and borderline groups. The overall mortality rate was 7.5% and showed a significant association with histological grade; mortality was 23.5% in the malignant group, 10.0% in the borderline group, and absent in the benign group (*p* < 0.05) (Table 1).

To further characterize the clinical significance of IMP3 expression, we analyzed its association with demographic and clinicopathological variables (Table 2). IMP3-positive tumors were significantly larger than IMP3-negative tumors (median: 7.0 cm versus 4.0 cm; *p* = 0.036). Stromal atypia was markedly more prevalent in IMP3-positive cases (72.2% versus 24.5%; *p* < 0.001). IMP3 expression showed a strong association with histological grade (*p* < 0.001): 68.4% of IMP3-positive tumors were malignant, compared with only 8.2% in the IMP3-negative group. Patients with IMP3-positive tumors tended to be older (median: 46.3 versus 37.6 years; *p* = 0.052) and had higher Ki-67 indices (median: 20.0% versus 10.0%; *p* = 0.061), although these differences did not reach statistical significance. No significant associations were observed between IMP3 expression and mitotic activity (*p* = 0.924), menopausal status (*p* = 0.310), or positive surgical margin status (*p* = 0.072).

The unadjusted Kaplan–Meier survival analyses demonstrated significant differences in OS according to histological grade and IMP3 expression in patients with PTs (Table 3). OS was significantly shorter in patients with malignant tumors compared with those with benign and borderline tumors (log-rank *p* = 0.009), with progressively lower 5-year OS rates observed across increasing histological grades.

In addition, in unadjusted Kaplan–Meier analysis, IMP3-positive tumors were associated with significantly reduced OS compared with IMP3-negative tumors (log-rank *p* = 0.013). Patients with IMP3 positivity exhibited lower mean survival times and inferior 5-year OS rates.

**Figure 3:**
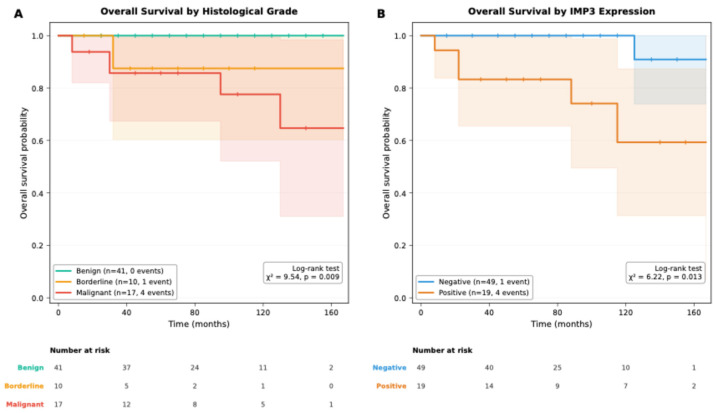
Kaplan–Meier overall survival curves in patients with PTs. (**A**) Overall survival stratified by histological grade: benign (*n* = 41, 0 events), borderline (*n* = 10, 1 event), and malignant (*n* = 17, 4 events). The difference among groups was statistically significant (log-rank χ^2^ = 9.54, *p* = 0.009). (**B**) Overall survival stratified by IMP3 expression status: negative (*n* = 49, 1 event) and positive (*n* = 19, 4 events). IMP3-positive tumors demonstrated significantly worse overall survival (log-rank χ^2^ = 6.22, *p* = 0.013). Numbers at risk are displayed below each curve. Shaded areas represent 95% confidence intervals. Abbreviations: IMP3, insulin-like growth factor II mRNA-binding protein 3.

Figure 3 illustrates unadjusted Kaplan–Meier OS curves stratified by histological grade and IMP3 expression. OS differed significantly according to histological grade, with malignant PTs exhibiting poorer survival compared with benign and borderline tumors (log-rank χ^2^ = 9.54; *p* = 0.009). In addition, IMP3-positive tumors were associated with significantly reduced OS compared with IMP3-negative tumors (log-rank χ^2^ = 6.22; *p* = 0.013).

Based on unadjusted descriptive survival estimates, no statistically significant differences were observed in DFS according to histological grade, IMP3 status, or CD10 expression. Similarly, CD10 expression was not significantly associated with OS. These nonsignificant findings are detailed in Table 3. Univariate Cox regression analyses evaluating the association of treatment-related variables with OS and DFS are presented in Appendix A (Table A1 and Table A2).

## 4. Discussion

In this study, we demonstrated that IMP3 expression is strongly associated with histological grade and OS in PTs, supporting its role as a promising adjunct biomarker in this rare disease. IMP3 positivity was detected in 76.5% of malignant tumors but in only 7.3% of benign tumors. Notably, 80% of the deceased patients had IMP3-positive PTs. Although these associations, derived from descriptive comparisons and survival analyses, suggest a link between IMP3 expression and aggressive tumor behavior; they do not establish independent prognostic significance.

**Figure A1:**
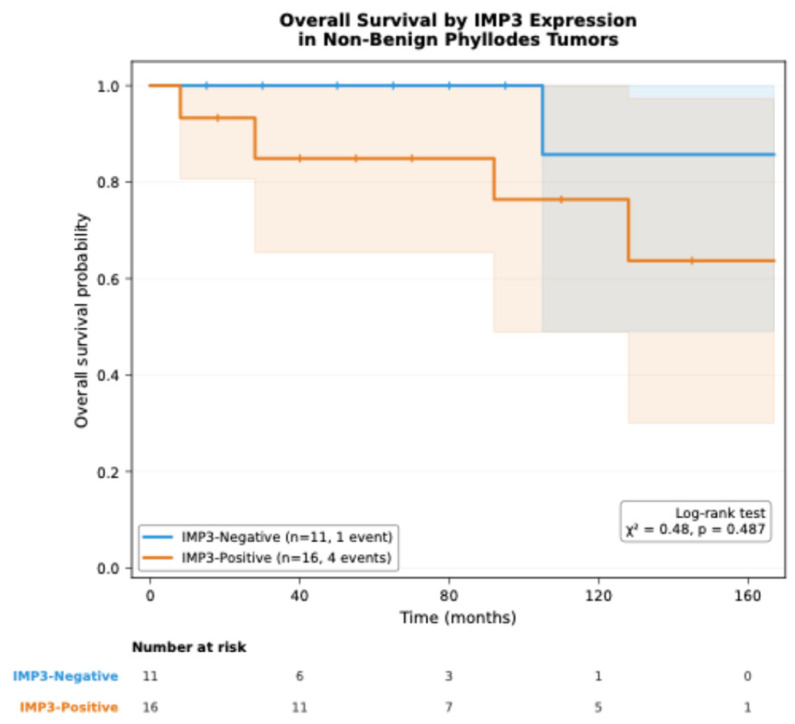
Kaplan–Meier overall survival curves stratified by IMP3 expression in non-benign phyllodes tumors. The analysis was restricted to borderline and malignant tumors (*n* = 27) to evaluate whether IMP3 provides prognostic information within histological grade categories. IMP3-positive tumors (*n* = 16, 4 events) demonstrated numerically lower survival compared with IMP3-negative tumors (*n* = 11, 1 event); however, the difference did not reach statistical significance (log-rank χ^2^ = 0.48, p = 0.487). Numbers at risk are displayed below the curve. Shaded areas represent 95% confidence intervals. Abbreviations: IMP3, insulin-like growth factor II mRNA-binding protein 3.

IMP3 is a well-established oncofetal protein implicated in tumor progression and consistently linked to poor clinical outcomes across multiple malignancies [26]. It enhances metastatic potential through induction of epithelial-mesenchymal transition [27], and promotes tumorigenesis via post-transcriptional regulation of IGF-II mRNA translation, integrating mRNA stability with oncogenic signaling pathways [28]. In line with its pro-tumorigenic role, IMP3-positive tumors in our cohort exhibited more aggressive clinicopathological features, including higher grade, larger tumor size, and increased stromal atypia. These findings are in accordance with those of Bellezza et al., who reported strong stromal IMP3 positivity in 56% of malignant PTs and demonstrated higher expression in recurrent tumors [21]. However, in the present study, the observed association between IMP3 positivity and mortality was based solely on group-level comparisons and Kaplan–Meier survival analyses. Because of the very low number of outcome events (*n* = 5 deaths), regression-based risk estimation was not performed. Therefore, the data do not allow assessment of whether IMP3 provides prognostic information independent of histological grade. A supplementary Kaplan–Meier analysis restricted to non-benign (borderline and malignant) tumors did not demonstrate a statistically significant difference in OS according to IMP3 status (log-rank *p* = 0.487; Figure A1).

CD10-positive patients exhibited lower 5-year DFS rates than CD10-negative patients (60.5% versus 88.6%, respectively); however, the difference did not translate into a statistical significance. Similarly, OS was comparable between the same groups. In PTs, CD10 expression is primarily confined to the stromal component and is associated with higher histological grade and invasive growth patterns, suggesting a role in stromal activation and tumor aggressiveness [29]. While our results do not fully support a prognostic role for CD10, they indicate the need for validation in larger cohorts before definitive conclusions can be drawn.

In our study, pathological grade emerged as the strongest predictor of OS (*p* = 0.009), with a mortality rate of 23.5% in the malignant group compared with no deaths in the benign group.

Our study has several strengths, including a homogeneous cohort from a single institution and comprehensive immunohistochemical evaluation. To our knowledge, this is among the first studies to evaluate IMP3 and CD10 together with survival endpoints in a Turkish PT cohort.

Nevertheless, this study also has several limitations. First, the single-center, retrospective design may limit the generalizability of the findings. In addition, the very low number of outcome events, particularly deaths (*n* = 5), substantially constrained statistical inference, precluding regression-based analyses and formal evaluation of independent prognostic values of IMP3 or CD10 beyond other clinicopathological variables. Another important limitation is the long inclusion period spanning more than two decades (2000–2024). Although this extended timeframe was necessary due to the rarity of PTs, it may have introduced temporal heterogeneity that could influence survival outcomes independently of tumor biology. Consequently, the observed associations—particularly those involving IMP3 expression and OS—may partly reflect treatment-era or management-related differences rather than intrinsic prognostic effects alone. Given the limited number of outcome events, comprehensive stratified or sensitivity analyses according to treatment modalities (such as radiotherapy or chemotherapy) were not feasible, and these potential confounding effects could not be fully addressed.

Furthermore, all histopathological and immunohistochemical evaluations were performed by a single, experienced pathologist. Although this approach ensured internal consistency in slide interpretation and scoring, it precluded assessment of interobserver variability. Although the scoring criteria used predefined cutoff values (>10% for IMP3 and >20% for CD10), cases with staining intensities near these thresholds may be subject to interpretation differences among observers. As a result, the reproducibility of IMP3 and CD10 expression assessment across different observers could not be evaluated, which may limit the applicability of the findings to broader pathology practice. Future multicenter studies incorporating independent or blinded multiobserver assessments would be valuable to confirm the robustness and reproducibility of these results.

Finally, treatment-related confounding represents an additional limitation. Surgical and adjuvant treatment modalities were more frequently applied in patients with aggressive disease, reflecting confounding by indication rather than true treatment effects. Given the descriptive nature of the analyses and the limited number of events, adjustment for treatment-related variables was not feasible. In addition, detailed information regarding surgical margin width was not consistently available due to the retrospective nature and long inclusion period. Therefore, margins were categorized dichotomously as positive or negative. Similarly, adjuvant treatment data were recorded as binary variables, and information on specific regimens, indications, or timing was incomplete, precluding regimen-specific analysis.

Overall, these limitations underscore the need for larger, prospective, multicenter studies with more uniform treatment protocols to clarify the prognostic relevance of IMP3 and CD10 and to determine whether these markers provide incremental value beyond established histopathological classification.

From a clinical standpoint, our findings do not support immediate changes in management or follow-up strategies based solely on IMP3 status. Rather, IMP3 should be viewed as a biologically plausible and potentially informative adjunct marker that may complement, but not replace, established histopathological risk stratification. Prospective, multicenter studies with larger event numbers are required to determine whether IMP3 provides incremental prognostic value beyond histological grade.

## 5. Conclusions

In conclusion, our study suggests that IMP3 expression is closely associated with higher histological grade and poorer OS in PTs, supporting its role as a promising adjunct prognostic marker. CD10 expression showed a nonsignificant trend toward shorter DFS and requires further evaluation. Although IMP3 appears biologically linked to aggressive tumor behavior, its incremental prognostic value beyond histological grade could not be definitively established in this cohort. Therefore, IMP3 should currently be considered a hypothesis-generating biomarker rather than a clinically actionable determinant, pending validation in larger, prospective, multicenter studies with sufficient outcome events.

## Figures and Tables

**Table 1:** Comparative analysis of clinicopathological characteristics and immunohistochemical marker expression patterns according to histological grade in patients with phyllodes tumors.

Variables	Total (*n* = 68)	Benign (*n* = 41)	Borderline (*n* = 10)	Malignant (*n* = 17)	*p*-Values
**Demographic characteristics**					
Age (years)	39.0 [13.9–74.0]	34.6 [13.9–70.6]	41.9 [15.6–62.5]	46.3 [20.7–74.0]	0.054
**Tumor characteristics**					
Tumor size (cm)	5.0 [1.0–14.0]	4.0 [1.0–12.0]	4.5 [1.5–9.0]	7.0 [2.0–14.0]	0.171
**Histopathological features**					
Mitotic activity					
<5/10 HPF	15 (37.5)	12 (48.0) ^b^	3 (60.0) ^b^	0 (0.0) ^a^	**0.005**
5–9/10 HPF	10 (25.0)	6 (24.0) ^a^	2 (40.0) ^a^	2 (20.0) ^a^	
≥10/10 HPF	15 (37.5)	7 (28.0) ^b^	0 (0.0) ^b^	8 (80.0) ^a^	
Ki-67 index (%)	15.0 [1.0–100.0]	10.0 [1.0–40.0] ^ab^	15.0 [3.0–20.0] ^b^	25.0 [10.0–100.0] ^a^	**<0.001**
Stromal atypia	25 (37.3)	9 (22.0) ^b^	2 (22.2) ^b^	14 (82.4) ^a^	**<0.001**
**Immunohistochemical markers**					
CD10 positivity	22 (32.4)	9 (22.0)	5 (50.0)	8 (47.1)	0.065
IMP3 positivity	19 (27.9)	3 (7.3) ^b^	3 (30.0) ^c^	13 (76.5) ^a^	**<0.001**
Estrogen receptor positivity	19 (46.3)	10 (58.8)	2 (28.6)	7 (41.2)	0.406
Progesterone receptor positivity	18 (43.9)	9 (52.9)	2 (28.6)	7 (41.2)	0.527
**Treatment characteristics**					
Surgical procedure					
Lumpectomy	61 (89.7)	39 (95.1)	8 (80.0)	14 (82.4)	0.219
Mastectomy	7 (10.3)	2 (4.9)	2 (20.0)	3 (17.6)	
Positive surgical margin	19 (29.2)	10 (24.4)	1 (12.5)	8 (50.0)	0.119
Postoperative radiotherapy	21 (31.8)	9 (23.1) ^b^	2 (20.0) ^b^	10 (58.8) ^a^	**0.022**
Adjuvant chemotherapy	6 (9.0)	1 (2.4) ^b^	2 (22.2) ^a^	3 (17.6) ^a^	**0.044**
**Clinical outcomes**					
Recurrence/Progression	12 (17.6)	6 (14.6)	1 (10.0)	5 (29.4)	0.435
Distant metastasis	3 (7.0)	0 (0.0)	1 (11.1)	2 (13.3)	0.212
Mortality	5 (7.5)	0 (0.0) ^b^	1 (10.0) ^a^	4 (23.5) ^a^	**0.008**

Data are presented as *n* (%) or median [minimum–maximum]. The Kruskal-Wallis H test was used for continuous variables, and the Pearson chi-square test or Fisher exact test was applied for categorical variables. When omnibus tests revealed significant differences, post-hoc pairwise comparisons were performed using Bonferroni-corrected tests. Different superscript letters (^a^, ^b^, ^c^) within the same row indicate statistically significant differences between groups; groups sharing the same superscript letter are not significantly different from each other. Bold *p*-values indicate statistical significance (*p* ≤ 0.05). Abbreviations: HPF, high-power field; IMP3, insulin-like growth factor 2 mRNA-binding protein 3; CD10, cluster of differentiation 10.

**Table 2:** Association of IMP3 expression with clinicopathological variables in patients with phyllodes tumors.

Variables	IMP3-Negative (*n* = 49)	IMP3-Positive (*n* = 19)	*p*-Value
**Age (years)**	37.6 [13.9–70.6]	46.3 [19.3–74.0]	0.052
**Tumor size (cm)**	4.0 [1.0–14.0]	7.0 [3.0–11.0]	**0.036**
**Ki-67 index (%)**	10.0 [1.0–40.0]	20.0 [2.0–100.0]	0.061
**Stromal atypia**, *present*	12 (24.5)	13 (72.2)	**<0.001**
**Mitotic activity**			
<5/10 HPF	11 (39.3)	4 (33.3)	0.924
5–9/10 HPF	7 (25.0)	3 (25.0)	
≥10/10 HPF	10 (35.7)	5 (41.7)	
**Histological grade**			
Benign	38 (77.6)	3 (15.8)	**<0.001**
Borderline	7 (14.3)	3 (15.8)	
Malignant	4 (8.2)	13 (68.4)	
**Menopausal status**			
Premenopausal	34 (69.4)	10 (52.6)	0.310
Postmenopausal	15 (30.6)	9 (47.4)	
**Positive surgical margin**	11 (22.9)	8 (42.1)	0.072

Data are presented as median [minimum–maximum] or *n* (%). Mann-Whitney U test was used for continuous variables and chi-square test or Fisher exact test for categorical variables. Bold *p*-values indicate statistical significance (*p* ≤ 0.05).

**Table 3:** Kaplan–Meier survival analysis according to histological grade and immunohistochemical marker expression in patients with phyllodes tumors.

	PT Classes				IMP3			CD10		
	Benign	Borderline	Malignant	*p*	Positive	Negative	*p*	Positive	Negative	*p*
**A. Disease-Free Survival**										
*n (events)*	41 (6)	10 (2)	17 (6)		19 (6)	49 (8)		22 (7)	46 (7)	
*Mean survival, months*	141.4	113.0	97.0	0.126	112.9	137.0	0.114	97.4	139.8	0.076
*5-year rate, % (95% CI)*	86.1 (69.4–94.0)	77.1 (34.5–93.9)	68.3 (39.5–85.5)		65.9 (39.1–83.1)	85.8 (70.8–93.5)		60.5 (33.4–79.4)	88.6 (74.7–95.1)	
**B. Overall Survival**										
*n (events)*	41 (0)	10 (1)	17 (4)		19 (4)	49 (1)		22 (2)	46 (3)	
*Mean survival, months*	163.0	126.9	130.3	**0.009**	132.8	158.4	**0.013**	152.0	153.1	0.573
*5-year rate, % (95% CI)*	100.0 (—)	87.5 (38.7–98.1)	86.9 (56.5–96.6)		82.9 (55.7–94.2)	100.0 (—)		89.2 (63.1–97.2)	97.8 (85.6–99.7)	

Survival estimates were calculated using the Kaplan–Meier method, and between-group comparisons were performed using the log-rank test. Median survival was not reached in any subgroup; therefore, mean survival times are reported. Bold *p*-values indicate statistical significance (*p* ≤ 0.05). Abbreviations: CI, confidence interval; IMP3, insulin-like growth factor II mRNA-binding protein 3; CD10, cluster of differentiation 10; PT, phyllodes tumor.

## Data Availability

The data presented in this study are available on request from the corresponding author due to privacy and ethical restrictions.

## Abbreviations

The following abbreviations are used in this manuscript:

CD10	Cluster of differentiation 10
CI	Confidence interval
CT	Computed tomography
DFS	Disease-free survival
EGFR	Epidermal growth factor receptor
ER	Estrogen receptor
EZH2	Enhancer of zeste homolog 2
FFPE	Formalin-fixed, paraffin-embedded
H3K27me3	Trimethylation of histone H3 at lysine 27
HPF	High-power field
IMP3	Insulin-like growth factor II mRNA-binding protein 3
MRI	Magnetic resonance imaging
OS	Overall survival
PD-L1	Programmed death-ligand 1
PR	Progesterone receptor
PRC2	Polycomb repressive complex 2
PT	Phyllodes tumor
SD	Standard deviation
TAM	Tumor-associated macrophage
WHO	World Health Organization

## Appendix A

**Table A1:** Univariate Cox Regression Analysis of Treatment-Related Variables for Overall Survival and Disease-Free Survival.

Outcomes	Variables	*n*	Events	HR	95% CI	*p*-Value
**Overall Survival**						
	Mastectomy vs. Lumpectomy	67	5	6.49	1.46–28.77	**0.014**
	Postoperative Radiotherapy	66	5	0.71	0.21–2.46	0.589
	Adjuvant Chemotherapy	67	5	8.32	1.85–37.34	**0.006**
**Disease-Free Survival**						
	Mastectomy vs. Lumpectomy	67	14	2.42	0.72–8.13	0.154
	Postoperative Radiotherapy	66	14	1.44	0.59–3.52	0.429
	Adjuvant Chemotherapy	67	14	2.85	0.83–9.74	0.095

Cox regression analyses were performed using L2-penalized maximum likelihood estimation due to the low number of events. Bold *p*-values indicate statistical significance (*p* ≤ 0.05). Abbreviations: CI, confidence interval; HR, hazard ratio.

**Table A2:** Event Distributions in Treatment Groups.

Variables	No	Yes	Deaths (Yes Group)	Recurrences (Yes Group)
Mastectomy	61	6	3/6 (50%)	3/6 (50%)
Postoperative RT	45	21	1/21 (4.8%)	6/21 (28.6%)
Adjuvant Chemotherapy	61	6	3/6 (50%)	3/6 (50%)
